# Supplementary material for: The IHAT-GUT Iron Supplementation Trial in Rural Gambia: Barriers, Facilitators, and Benefits
Source: Nutrients. 2021 Mar 30;13(4):1140. doi: 10.3390/nu13041140 (PMC8066312; doi:10.3390/nu13041140)
Supplement: Supplementary file 1 [file nutrients-13-01140-s001.pdf]

Supplementary Data

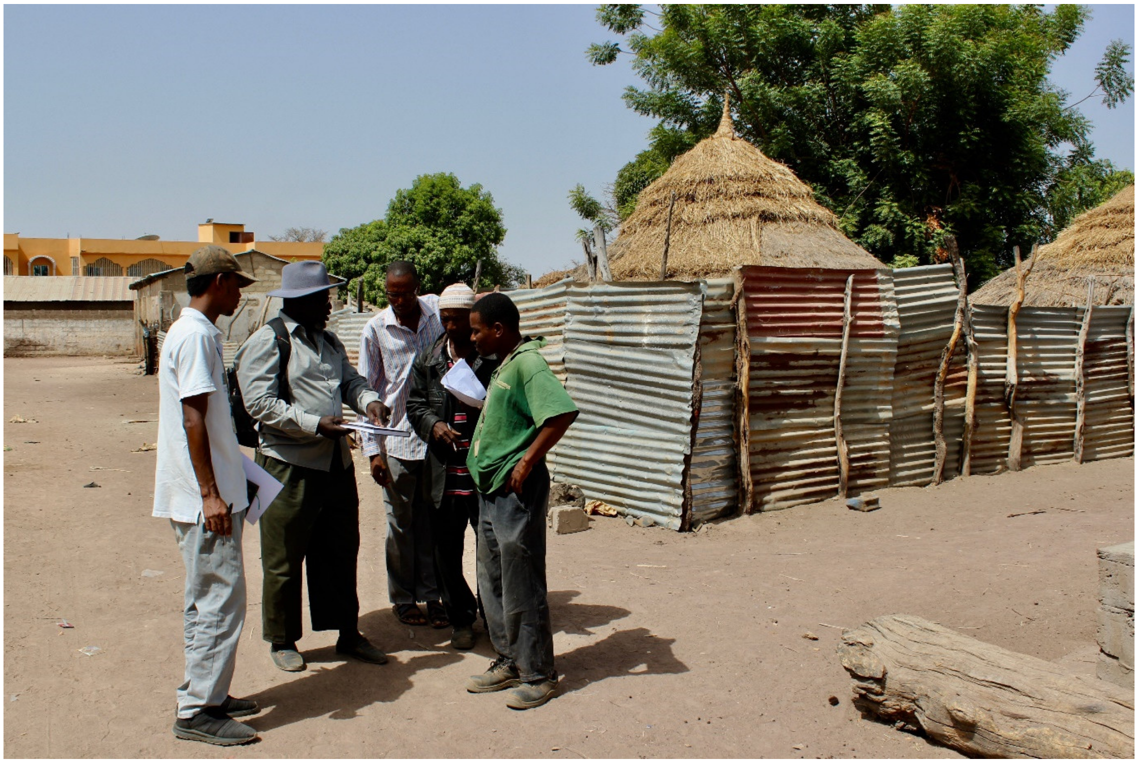

Figure S1. Photograph of members of the IHAT-GUT field team.

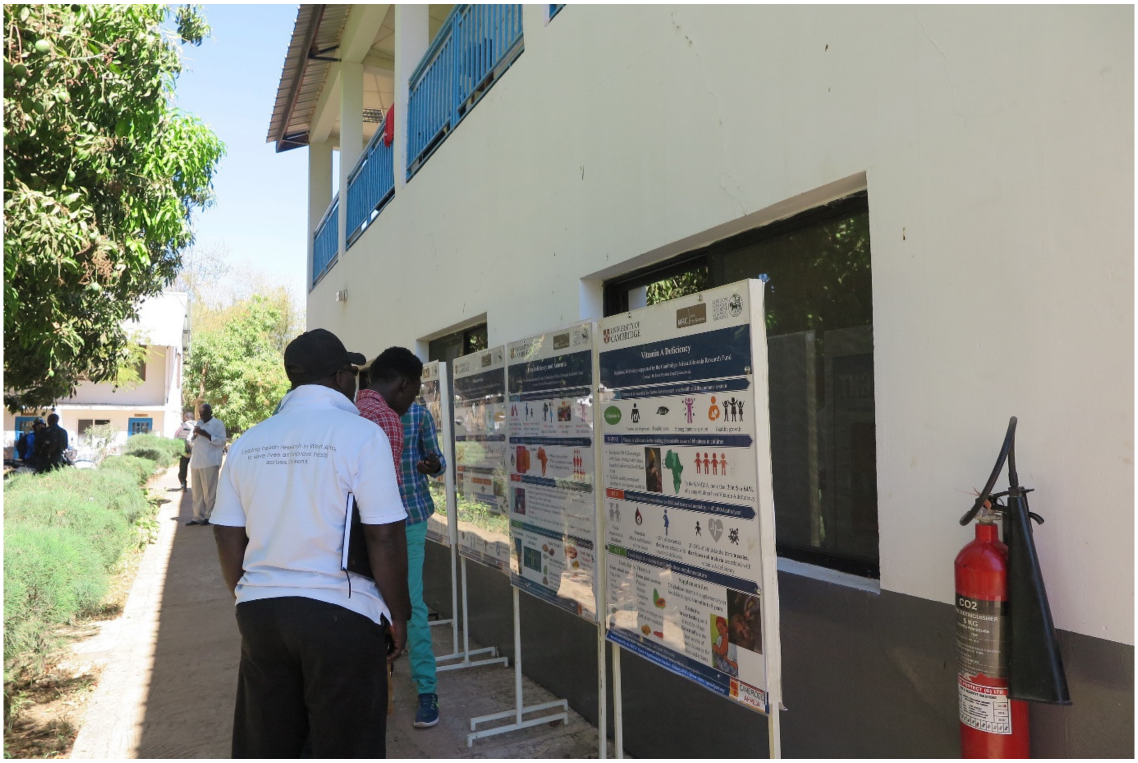

Figure S2. Photograph of the poster session at the Nutrition Workshop.

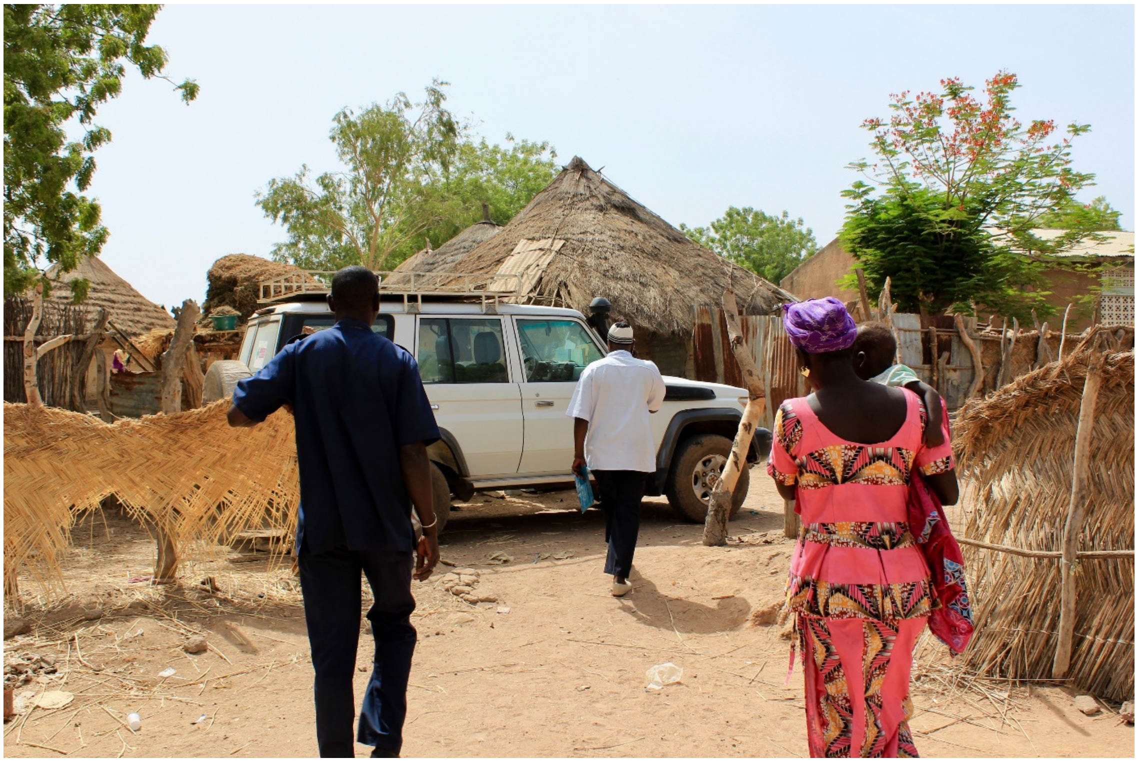

Figure S3. Photograph of Driver and Nurse Coordinator picking up a participant and mother from their home for evaluation of an adverse event.
